# A coarse-to-fine point completion network with details compensation and structure enhancement

**DOI:** 10.1038/s41598-024-52343-6

**Published:** 2024-01-23

**Authors:** Yongwei Miao, Chengyu Jing, Weihao Gao, Xudong Zhang

**Affiliations:** 1 School of Information Science and Technology, Hangzhou Normal University, Hangzhou, 311121 China; 2 School of Computer Science and Technology, Zhejiang Sci-Tech University, Hangzhou, 310018 China; 3 School of Information Science and Technology, Zhejiang Shuren University, Hangzhou, 310015 China

**Keywords:** Computer science, Mechanical engineering

## Abstract

Point cloud completion, the issue of estimating the complete geometry of objects from partially-scanned point cloud data, becomes a fundamental task in many 3d vision and robotics applications. To address the limitations on inadequate prediction of shape details for traditional methods, a novel coarse-to-fine point completion network (DCSE-PCN) is introduced in this work using the modules of local details compensation and shape structure enhancement for effective geometric learning. The coarse completion stage of our network consists of two branches—a shape structure recovery branch and a local details compensation branch, which can recover the overall shape of the underlying model and the shape details of incomplete point cloud through feature learning and hierarchical feature fusion. The fine completion stage of our network employs the structure enhancement module to reinforce the correlated shape structures of the coarse repaired shape (such as regular arrangement or symmetry), thus obtaining the completed geometric shape with finer-grained details. Extensive experimental results on ShapeNet dataset and ModelNet dataset validate the effectiveness of our completion network, which can recover the shape details of the underlying point cloud whilst maintaining its overall shape. Compared to the existing methods, our coarse-to-fine completion network has shown its competitive performance on both chamfer distance (CD) and earth mover distance (EMD) errors. Such as, the repairing results on the ShapeNet dataset of our completion network are reduced by an average of $35.62\%$, $33.31\%$, $29.62\%$, and $23.62\%$ in terms of CD error, comparing with PCN, FoldingNet, Atlas, and CRN methods, respectively; and also reduced by an average of $15.63\%$, $1.29\%$, $64.52\%$, and $62.87\%$ in terms of EMD error, respectively. Meanwhile, the completion results on the ModelNet dataset of our network have an average reduction of $28.41\%$, $26.57\%$, $20.65\%$, and $18.55\%$ in terms of CD error, comparing to PCN, FoldingNet, Atlas, and CRN methods, respectively; and also an average reduction of $21.91\%$, $19.59\%$, $43.51\%$, and $21.49\%$ in terms of EMD error, respectively. Our proposed point completion network is also robust to different degrees of data incompleteness and model noise.

## Introduction

Nowadays, due to its easy capturing, direct representation, and its ability to represent complex 3d shapes, point cloud data has received widespread attention and is widely applied in various model industries, such as intelligent robotics^1^, autonomous driving^2^, SLAM^3^ and augmented reality^4^, etc. However, for acquiring the discrete point cloud data of 3d shapes using a laser scanner or depth camera^5^, the captured 3d point cloud data is usually incomplete or has artificial defects due to its obstacle occlusion or light reflection during 3d scanning, which leads to some difficulties for the subsequent modeling and shape processing. For example, for intelligent robotics, the incomplete point cloud data of 3d objects usually make it difficult to recognize the target object to be grasped correctly and also difficult to perform accurate localization and grasping. In the applications of SLAM and augmented reality, the missing point cloud data often leads to being unable to correctly object detection and recognition, thus making it difficult to perform accurate target localization and achieve SLAM mapping.

Point cloud completion, which aims to recover the complete shapes from incomplete point clouds^6^, has attracted lots of interest since the high-quality reconstruction of complete shapes is always essential for the tasks of point cloud recognition^7^, point cloud simplification^8^, scene segmentation^9^ and 3d reconstruction^10^, etc. The traditional point completion network (PCN)^11^ employed a simple encoder–decoder framework to generate the complete shapes from incomplete point cloud data, and adopted FoldingNet operation for mapping 2d grid to 3d surfaces by mimicking the planar folding deformation^12^. However, traditional methods always have some difficulties in synthesizing the finer-grained details of point cloud shapes. The surface fine details (e.g., fine feature lines or thin surface patches of complex shapes) always have a sparse distribution of sampling points, which may not be effectively repaired using traditional networks due to their decoding into a complete point cloud data from a global feature vector^13^. With the help of atlas representation of 3d shapes and their manifold definition, the AtlasNet network introduced by Groueix et al.^14^ can partially approximate the target shapes by mapping a set of squares onto point set surfaces of the underlying objects. Wang et al.^15^ proposed a cascaded refinement network (CRN), which exploited a coarse to fine completion strategy for generating point clouds, and can obtain the dense complete point clouds through the cross-layer concatenate for the multiplied point clouds, shape features, and global features.

**Figure 1:**
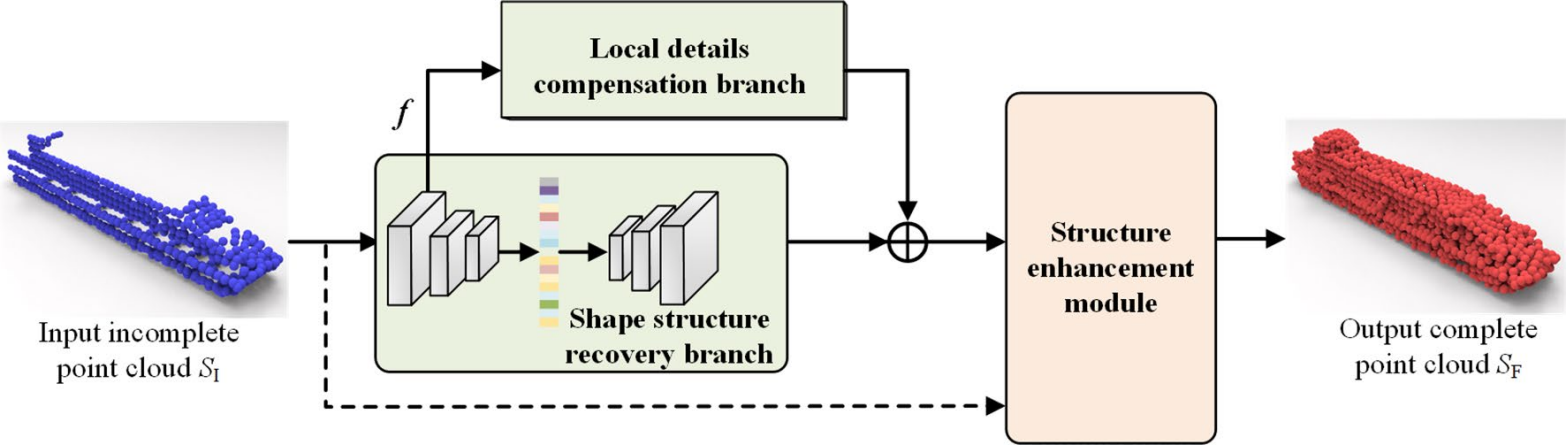
Network architecture of the proposed point completion network DCSE-PCN with local details compensation and shape structure enhancement.

For the traditional point complete networks, a global feature vector is generated from the input missing point cloud data through an encoder module, which is then decoded and transformed into complete point cloud data. These solutions will always neglect the learning of surface detail information in the original input shapes, thus resulting in a rough point cloud completion result with insufficient detail recovery. To overcome this limitation, we propose a novel coarse-to-fine point completion network (DCSE-PCN) in this work by introducing the modules of local details compensation and global structure enhancement for effective geometric learning. In the coarse completion stage, the network learns the geometric features and fuses the hierarchical features to recover its overall shape and also the shape details of raw point cloud data, which obtains a rough recovered point cloud. The fine completion stage of our network introduces the structure enhancement module to reinforce the correlated shape structures, thus obtaining the completed geometric shape with finer-grained details (see Fig. 1).

The main technical contributions of this work can be summarized as follows,

- We propose an end-to-end point completion network DCSE-PCN with local details compensation and shape structure enhancement for effective geometric learning thus to achieve coarse-to-fine point cloud completion.
- We design a network branch of local details compensation by employing feature learning and hierarchical feature fusion thus to maintain the shape details of the original incomplete point cloud.
- We introduce a structure enhancement module to reinforce the intrinsic structures through the learning of multi-scale point features and exploiting the self-attentive mechanism to infer the correlation structure of regular arrangement or symmetry, which can benefit to obtain the completed geometric shape with finer-grained details for the task of point cloud completion.

The rest of this paper is organized as follows. In “Related work”, we briefly overview some related works. The details of our presented point completion network DCSE-PCN are given in “Method”, including the overall network architecture and key modules. Experimental results are presented in “Experiments”. “Conclusion” summarizes the research content.

## Related work

### Multi-scale feature extraction

For the task of image classification, the traditional convolutional neural networks can effectively extract the image features through three different types of layers—the convolutional layer, pooling layer, and fully-connected layer^16^. To solve the degradation problem for the network training that arises from the deepening of network layers, He et al.^17^ introduced a residual network to improve the image recognition accuracy by optimizing the residual mapping. However, due to its irregular point cloud distribution, traditional convolutional operations cannot be directly applied to 3d point cloud data for feature extraction. Most existing networks can extract feature information from point clouds through pooling and flexible convolution operations. Qi et al. introduced PointNet^18^ to learn the point-wise features with multi-layer perceptron layers and extract the global shape features with a max-pooling layer. Subsequently, PointNet++^19^ improved the traditional PointNet^18^ by using a multi-scale downsampling structure to expand the receptive field between the input sampling points, which can learn local features with the increasing contextual scales, resulting in greatly decreased performance for networks trained on uniform densities. Owing to two point set abstraction layers, PointNet++^19^ can effectively aggregate multi-scale features according to local point densities thus improving different tasks of point cloud processing. Wu et al.^20^ proposed a convolution operation called Pointconv, which extends the traditional image convolution to point clouds and improves its performance by efficiently computing the weight function. As the voxel-based feature extraction scheme, Choy et al.^21^ presented a fully-convolutional geometric feature extraction module which can be computed in a single pass by a 3d fully-convolutional network. These features are compact, capture broad spatial context and scale to large scenes. Li et al. presented VoxFormer^22^, a Transformer based semantic scene completion network that can output complete 3D volumetric semantics from only 2D images, which utilizes the depth estimation to set voxel queries in a two-stage framework.

However, to overcome the issue of weakly capturing the intrinsic context information between long-distance sampling points, Ramachandran et al.^23^ employed self-attentive operations instead of common spatial convolution, thus improving the ability of convolution operations through a spatial-aware independent self-attentive layer. IMFNet introduced by Huang et al.^24^ is a multi-modal fusion method for point cloud feature extraction by considering structure and texture information. Moreover, by using a large vision pre-trained model (named as CLIP) as a point cloud encoder, Huang et al.^25^ presented an efficient point cloud learning module which is an effective point cloud learner for directly training high-quality point cloud models with a frozen CLIP transformer.

### Point completion networks

To efficiently repair the incomplete point cloud data, the traditional PCN network^11^ can generate a complete point cloud model from the global features learned from the partial input 3d shape. TopNet presented by Tchapmi et al.^26^ can predict the complete point cloud shape through a tree-structured decoder. Xie et al.^27^ proposed a gridding residual network (GRNet), which can transform the input point cloud data into 3d mesh models without loss of geometric information through mesh inversion, and thus effectively extract the contextual information through a 3d convolutional network. To generate shape details of 3d objects, Wang et al.^15^ designed a cascaded refinement network to synthesize the detailed object shapes together with a coarse-to-fine strategy, which can preserve the local details in the incomplete point cloud and recover the missing parts with high fidelity. Huang et al.^28^ proposed a point fractal network (PF-Net), which can preserve the spatial arrangement of incomplete shapes and also estimate the missing point cloud hierarchically by utilizing a feature points based multi-scale generating network. The variational relational completion network (VRCNet) presented by Pan et al.^29^ employed the probabilistic modeling through a dual-path network followed by a relational enhancement network and thus can effectively exploit 3d structural relations to predict the complete shapes. Using a simple encoder–decoder architecture, Miao et al.^30^ proposed an end-to-end point completion network that contains an encoder to encode information from neighboring points as well as a decoder to output dense complete point clouds.

However, the traditional point completion methods always neglect the learning of shape details due to their learning of global latent features through an encode–decode network framework. In this work, we propose a novel point completion network by employing the modules of local detail compensation and shape structure enhancement for effective geometric learning. Our coarse-to-fine completion network can not only recover the global shape of the underlying point cloud but also compensate for the local shape details, and finally synthesize a complete point cloud model with details preservation.

## Method

### Network architecture of DCSE-PCN

To effectively preserve the global shape of the incomplete point cloud, with the help of a self-attentive mechanism, we propose a novel coarse-to-fine completion network (DCSE-PCN) with details compensation and structure enhancement, as shown in Fig. 1. To effectively recover the shape details of the point cloud whilst maintaining its overall shape, the coarse completion stage of our network is to generate the rough recovered point cloud through two network branches—an encoder–decoder based branch for shape structure recovery and a details compensation branch. Here, the network branch for shape structure recovery can encode the raw point cloud into a feature code-word in the low-dimensional latent space through feature learning and hierarchical feature fusion, and thus recover the global shape by decoding the condensed latent feature information. However, this low-dimensional projection will extract semantic features to guide the global completion of the incomplete data, its local shape details observed from partial scanning data may often be lost. The network branch for details compensation takes the multi-level features from the encoder as input and performs the multi-granularity feature learning, which can recover the shape details through a detail reshaping operation. Furthermore, the fine completion stage employs the structure enhancement module to reinforce the correlated shape structures and thus obtain the complete geometric shape with finer-grained details.

The loss function introduced in our point completion network DCSE-PCN takes into account the CD error^31^ and EMD error^32^ between the first stage rough recovered point cloud and the ground truth, as well as the CD and EMD distance error between the second stage completion point cloud and the ground truth.

### Coarse completion stage with details compensation

**Figure 2:**
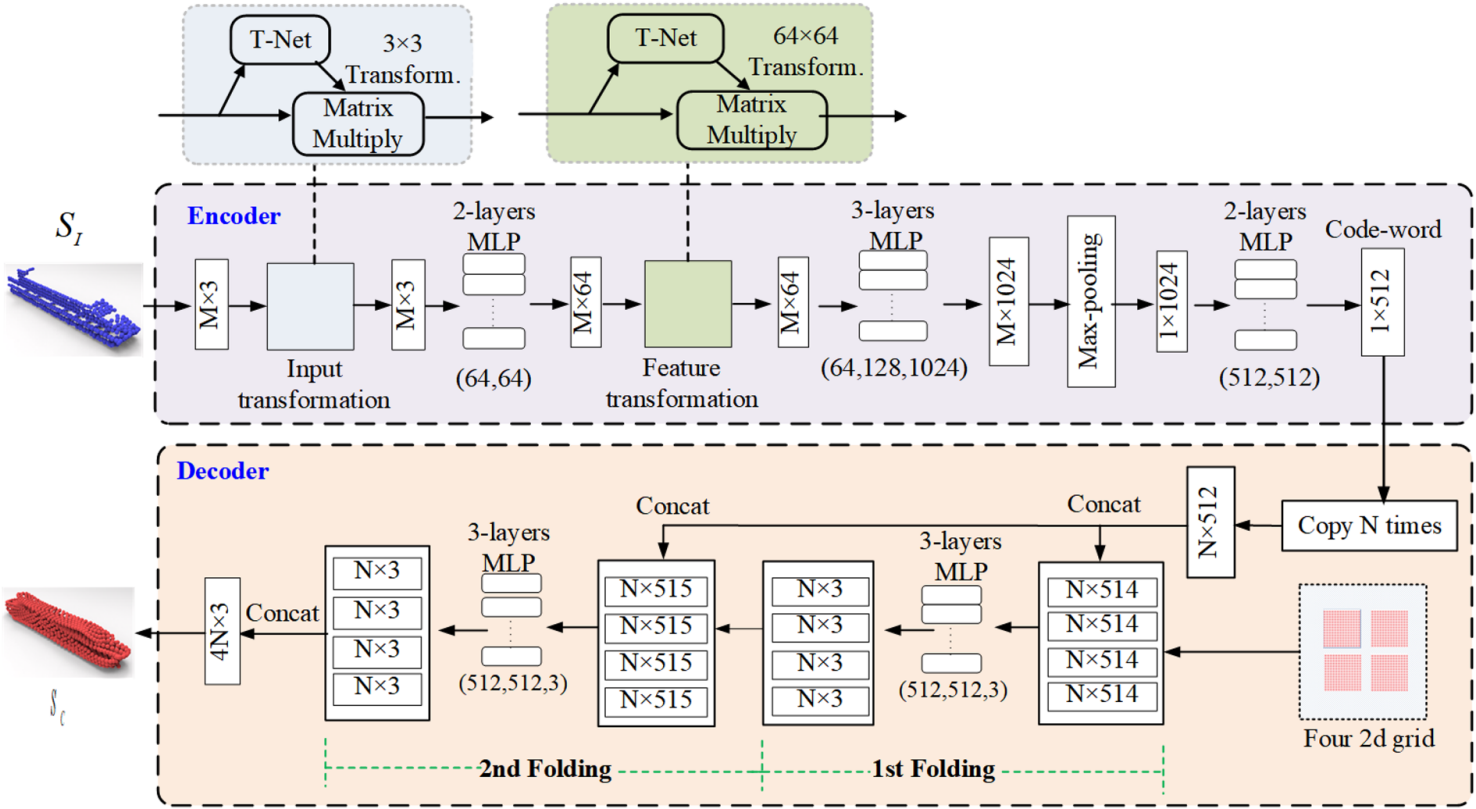
Shape structure recovery branch using an encoder–decoder framework.

To generate the complete shape with finer-grained details, we design an encoder–decoder framework to recover the global shape created from the encoded code-word in the coarse completion stage and also preserve the shape details as much as possible using the details compensation branch, which can then be employed as the input to the structure enhancement module in the fine completion stage for shape detail enhancement operation.

The coarse completion stage of the proposed network is a two-channel network architecture—a shape structure recovery branch and a local detail compensation branch. Here, the shape structure recovery branch employs an encoder–decoder structure (see Fig. 2). The encoder can create the feature code-word with global shape information through multi-layer perceptrons and max-pooling operation, while the folding-based decoder exploits four 2d grids for follow-up folding operations on the encoded code-word to reconstruct the coarse complete point cloud surface. The purpose of the local details compensation branch is to recover shape details of the original shape through multi-level feature extraction and feature fusion. The output of these two branches is finally stitched together and re-sampled to generate a coarse completion point cloud shape. In detail, as shown in Fig. 2, the input of the shape structure recovery branch is a $M\times 3$ matrix with the coordinates of input *M* discretely sampled points and embeds them to high-dimensional feature space with a 512-dimensional code-word. This code-word is the eigenvector extracted by the encoder which implicitly represents the global features of the input shape. With this 512-dimension code-word, our decoder can output a coarse completion shape with 4*N* sampling points through two sequential folding operations^12^ which deforms four 2d grids onto the underlying 3d surface of a point cloud.

**Figure 3:**
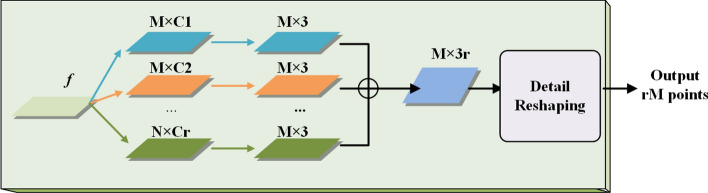
Local details compensation branch.

For the local details compensation branch (see Fig. 3), inspired by the hierarchical feature learning and multi-level feature fusion introduced by PU-Net network^34^, we employ the multi-level features obtained from the encoder and perform the multi-granularity feature learning, thus expanding the set of sampling points in the feature space and recovering the shape details using a detail reshaping operation. As shown in Fig. 3, the detail compensation branch firstly inputs the multi-scale features $M\times C_i$, $i=1,2,3,\ldots , r$ extracted from the encoder and then performs hierarchical feature learning, which can effectively extract the intrinsic geometric features of the underlying point cloud and obtain $M\times 3$ dimensional intermediate features. Furthermore, the $M\times 3r$ dimensional shape tensor feature can be created by stitching these *r* intermediate features, which can be converted into a point cloud with *rM* sampling points through a detail reshaping operation. Finally, we can obtain the synthesized point cloud data with $rM+4N$ sampling points by fusing both the output of the shape structure recovery branch and local details compensation branch and also re-sampled using IFPS^35^, which can generate a coarse complete shape that maintaining original shape details with uniform distribution of sampled points.

### Fine completion stage with structure enhancement

The purpose of shape completion is not only to preserve the global structure of the input point cloud model but also to maintain the fine details in the original shape based on correlational information. Inspired by the relational recognition of Zhao et al.^36^ for 2d images that use a self-attentive strategy, for the fine completion stage we further enhance the shape details of the generated coarse completion shape through the structure enhancement module which is based on the self-attentive operation and multi-scale point cloud feature learning.

**Figure 4:**
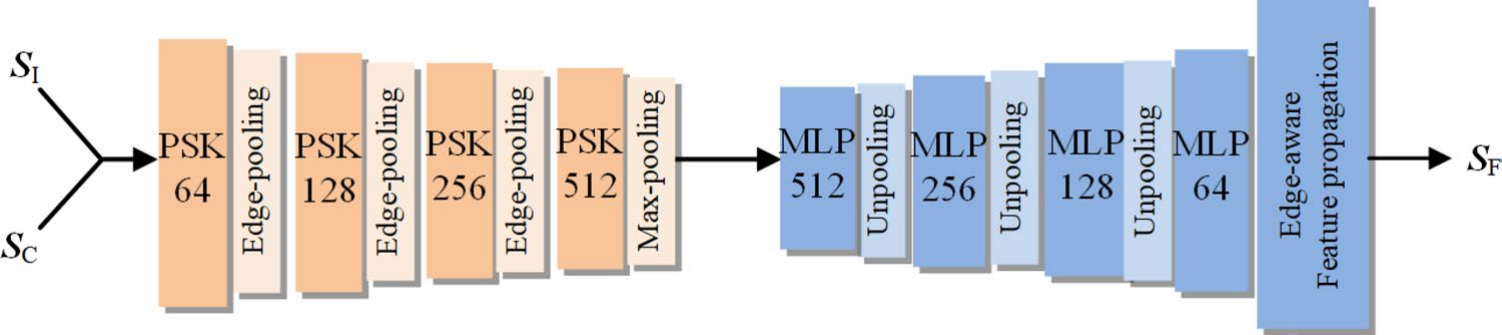
Structural enhancement module using hierarchical feature learning and multi-level feature fusion.

After obtaining a coarse repaired shape, the introduced structure enhancement module targets enhancing the correlated shape structures through the learning of multi-scale neighboring point features and thus adjusting the receptive fields to exploit and fuse multi-scale point features. As shown in Fig. 4, the structure enhancement module adopts a hierarchical encoder–decoder structure, which jointly inputs both the original incomplete point cloud and the coarse completion shape in the initial stage of our proposed network. In detail, our structure enhancement module exploits Point Selective Kernel module (PSK) and pooling operations at different scales to efficiently extract the shape details of the underlying point cloud and thus can generate feature latent vectors using the fully connected layers. This feature latent vector is fed to different dimensional multi-layer perceptrons and un-pooling operations to extract the edge-aware point features, and the fine complete shape can finally be generated using the edge-aware feature propagation operation to these edge-aware point features.

**Figure 5:**
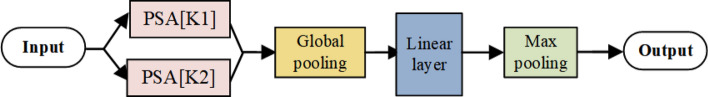
PSK module.

As shown in Fig. 5, our introduced PSK module is a multi-branching structure with different kernel sizes that can effectively utilize and fuse the multi-scale neighboring point features to further improve point cloud completion. Here, the purpose of Point Self-Attention kernel block (PSA) is to adaptively aggregate local neighboring point features with learned relations between neighboring points, which can be formulated as,

$$\begin{aligned} y_i=\sum _{j\in N(i)}{\alpha [x_{N(i)}]_j\cdot \beta (x_j)} \end{aligned}$$

where $x_{N(i)}$ is the group of point feature vectors for the selected K-nearest neighboring points *N*(*i*), and $\alpha [x_{N(i)}]$ is a weighting tensor for all selected feature vectors. $\beta (x_j)$ is the transformed features for point *j*, which has the same spatial dimensionality with $\alpha [x_{N(i)}]_j$. Afterward, we obtain the output $y_i$ using an element-wise product $\bullet$, which performs a weighted summation for all sampling points $j\in N(i)$.

### Loss function

For the task of point cloud completion, to effectively measure the difference between the completion results and the ground truth, and also to evaluate the performance of our proposed coarse-to-fine completion network, the Chamfer Distance (CD)^31^ and Earth Mover Distance (EMD)^32^ are always employed to measure the distance error.

Given a rough recovered point cloud $S_C$ and the ground truth $S_{GT}$, the CD distance error is defined as follows,

$$\begin{aligned} CD(S_C,S_{GT})=\frac{1}{|S_C|}\sum _{{\textbf {x}} \in S_C}min_{{\textbf {y}} \in S_{GT}}\Vert {\textbf {x}} -{\textbf {y}} \Vert _2 +\frac{1}{|S_{GT}|}\sum _{{\textbf {y}} \in S_{GT}}min_{{\textbf {x}} \in S_C}\Vert {\textbf {y}} -{\textbf {x}} \Vert _2 \end{aligned}$$

Similarly, we also can calculate CD distance error for a fine completed point clouds $S_F$ and the ground truth $S_{GT}$ as $CD(S_F, S_{GT})$. Here, $|S_F|$ and $|S_{GT}|$ denote the number of sampling points included the recovered point clouds and the ground truth respectively. The minimal distance error $CD(S_F, S_{GT})$ can make surfaces of recovered point clouds close to the ground truth, and also make recovered shapes covering all the missing parts.

In our loss function, the EMD distance error is computed as follows,

$$\begin{aligned} EMD(S_C,S_{GT})=min_{\phi :S_C\rightarrow S_{GT}}\frac{1}{|S_C|}\sum _{{\textbf {x}} \in S_C}\Vert {\textbf {x}} -\phi ({\textbf {x}} )\Vert _2 \end{aligned}$$

We implement EMD distance error based on finding a bijective function $\phi :S_C\rightarrow S_{GT}$, which make two point distributions close. In fact, we adopt Bertsekas’ scheme^33^ to compute the map $\phi$.

The loss function of our two-stage completion network can be calculated as follows,

$$\begin{aligned} L=\alpha [CD(S_C,S_{GT})+EMD(S_C,S_{GT})] + \beta [CD(S_F,S_{GT})+EMD(S_F,S_{GT})] \end{aligned}$$

The first item evaluates the result of “Coarse completion stage” for overall shape recovery, whilst the second loss item evaluates the result of “Fine completion stage” for structure enhancement. By minimizing this first item, we can make the global shape of the recovered point cloud close to the ground truth. By minimizing the second item, we can restore the elaborate shape structures. Here, we set the weight parameters $\alpha$ and $\beta$ satisfying $\alpha +\beta =1.0$. If we select a big $\alpha$, it may lead to uneven distribution of the sampling points. If we choose a big $\beta$, it may introduce noisy sampling points during the coarse-to-fine completion operation. In our experiments, we set $\alpha$ and $\beta$ equal to 0.5.

## Experiments

### Datasets and implementation

The proposed point completion network DCSE-PCN has been implemented on Ubuntu OS using Tensorflow and CUDA frameworks and a GPU of NVIDIA GTX 1080TI. The software platforms are Python 3.5.3 and Tensorflow 1.12.0. For the coarse completion stage, the shape structure and local details of the original incomplete point cloud can be effectively preserved using the two-branch completion network. And for the fine completion stage, the structure enhancement module can benefit to the generation of fine-grained complete point cloud through feature extraction and self-attention based relationship learning between neighboring points.

We train our coarse-to-fine completion network on ModelNet40 dataset^37^ and ShapeNet dataset^38^ including 4000 point cloud models randomly selected from each of dataset respectively. These models include various categories (40 categories from the ModelNet dataset and 8 categories from the ShapeNet dataset) of incomplete point clouds and their corresponding complete point clouds, such as airplanes, cars, ships, tables, beds, cones, benches, and chairs etc. We split 4000 objects into a train set (3000 objects) and a test set (1000 objects). The input of our point completion network is incomplete point clouds, and ground truth is corresponding complete models. All input point cloud data is centered at the (0, 0, 0), and their coordinates are normalized to $[-1, 1]^3$. The network training epoch is set to 100, which is trained by the ADAM optimizer^39^ with the learning rate of 0.0001 and batch size of 16.

### Completion results via DCSE-PCN

**Figure 6:**
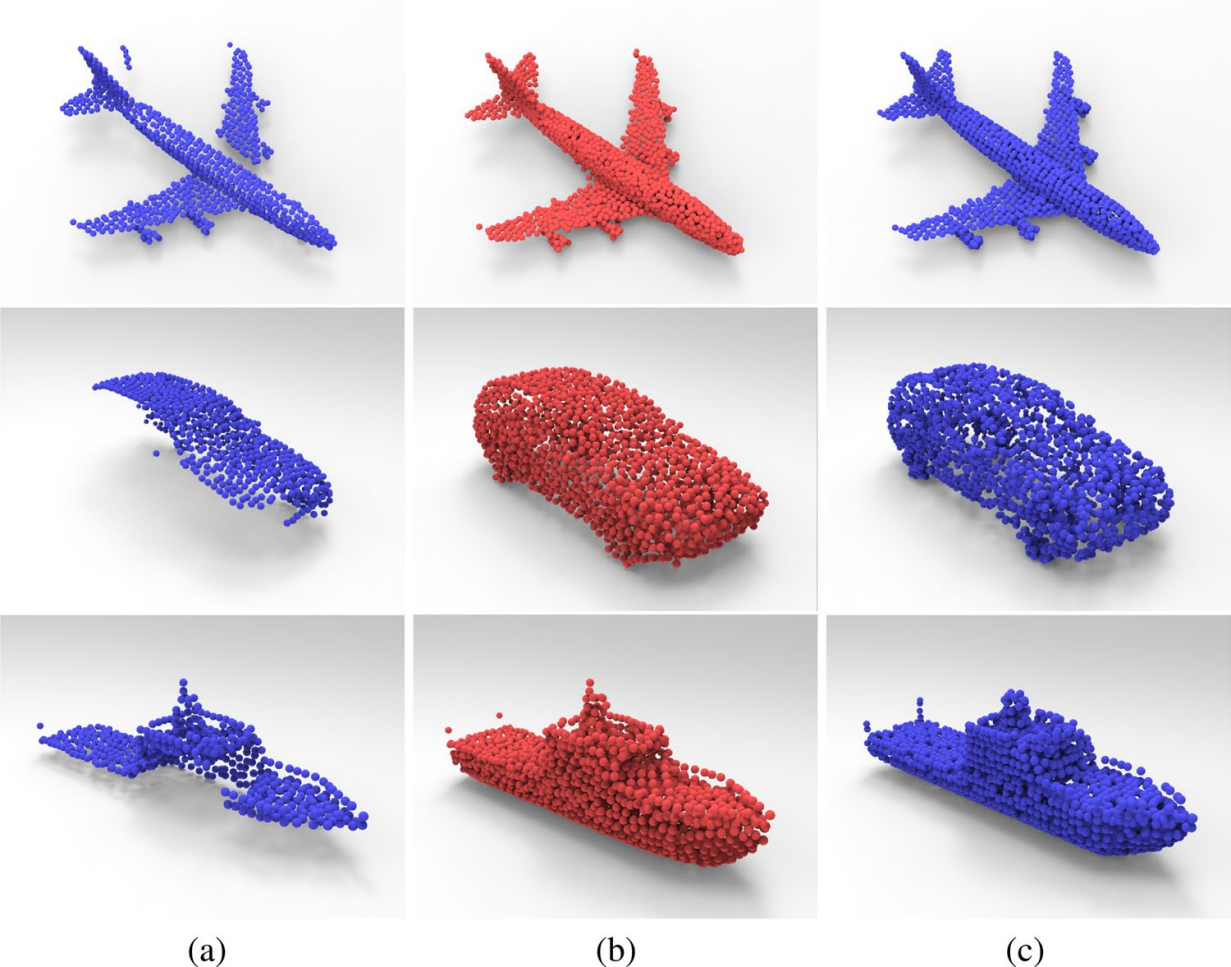
Visual performance of completion results via our presented completion network: (**a**) input incomplete point clouds, (**b**) shape completion results, (**c**) ground truth.

We propose a coarse-to-fine point completion network using hierarchical feature learning and a self-attentive mechanism. The performance of completion results is evaluated by the qualitative and quantitative results given in Fig. 6 and Table 1 respectively.

Figure 6 shows the visual performance of the completion results via our coarse-to-fine completion network. Figure 6a,c list the original incomplete point cloud and the corresponding ground truth, whilst Fig. 6b gives the complete point cloud shape repaired by our coarse-to-fine completion network. It can be seen that our presented network is able to repair the overall shapes of the input incomplete point cloud data, such as the missing tail and half of fuselage of airplane, the doors and tyres of car, the body and bottom of ship. Moreover, the original details and shape structures, such as the engine of airplane, the reversing mirror of car, and the components of ship body, can still be effectively maintained. The complete shape generated by our coarse-to-fine completion network is close to the corresponding ground truth, while the fine completion stage can produce uniform distributed sampling points. To evaluate our method, Table 1 gives the CD and EMD error via our coarse-to-fine completion network. The error statistics show that the CD and EMD error of the final completed point cloud through the structure enhancement module are always smaller than those of rough recovered shape in the coarse completion stage, which reflects the more uniform distribution of sampling points owing to the introduced structure enhancement module.

**Table 1:** Error statistics of different stages in our completion network.

Error type	Point cloud models	Coarse stage	Fine stage
CD	Airplane	0.01670	0.01501
	Car	0.02160	0.01935
	Watercraft	0.02243	0.02053
EMD	Airplane	0.04263	0.03163
	Car	0.03410	0.03201
	Watercraft	0.02633	0.02197

### Robustness to data missing and model noise

**Figure 7:**
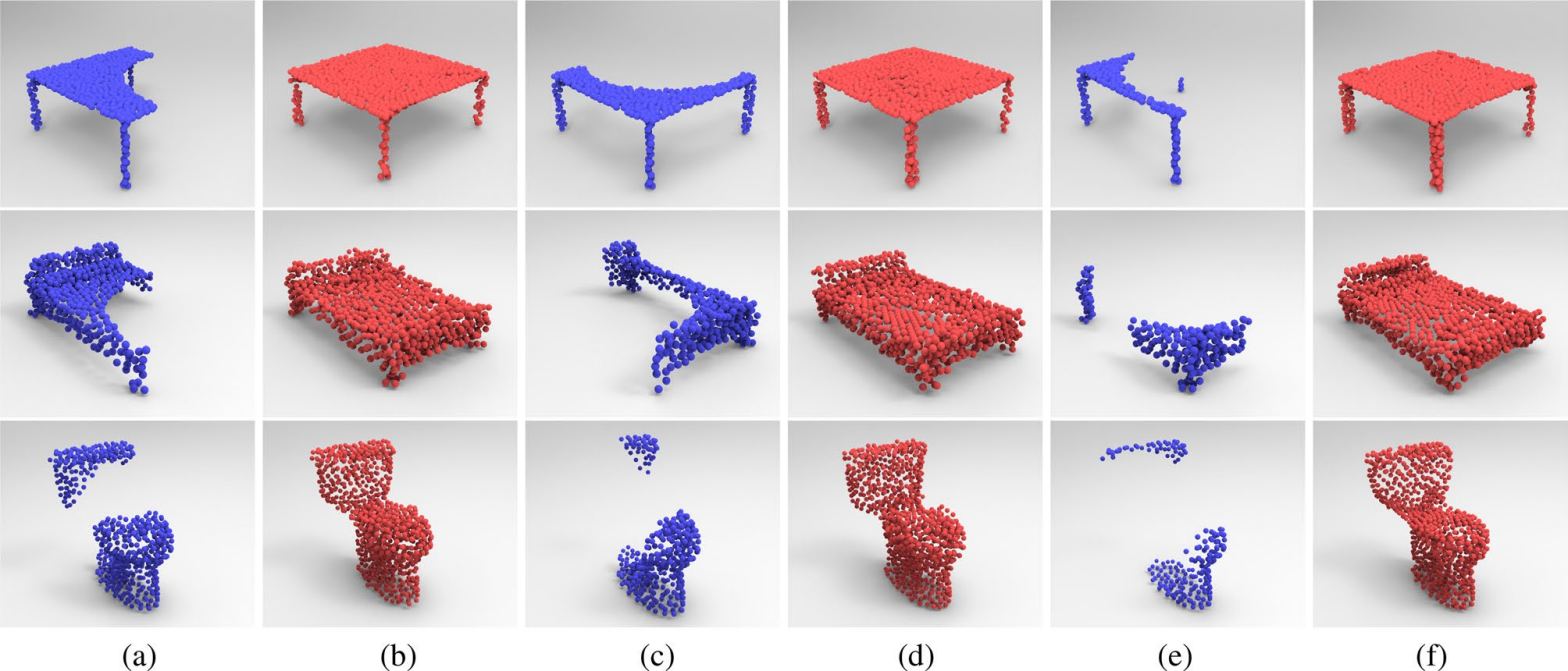
Visual performance of completion results with different degrees of data incompleteness: (**a**,**b**) list the input point cloud data with $40\%$ incompleteness and corresponding completion results. (**c**,**d**) List the input point cloud data with $60\%$ incompleteness and corresponding completion results. (**e**,**f**) List the input point cloud data with $80\%$ incompleteness and corresponding completion results.

To evaluate our presented coarse-to-fine point cloud completion method, we experiment with different degrees of data incompleteness and model noise. Figure 7 shows the completion results with $40\%$ (Fig. 7a), $60\%$ (Fig. 7c), and $80\%$ (Fig. 7e) sampling points missing. According to the corresponding completion results (see Fig. 7b,d,f respectively), it can be seen that the recovered point cloud model can preserve the global shape of the input data and also maintain its shape details even with a heavy data missing. The experimental results demonstrate that our network DCSE-PCN is robust against different degrees of data incompleteness.

**Figure 8:**
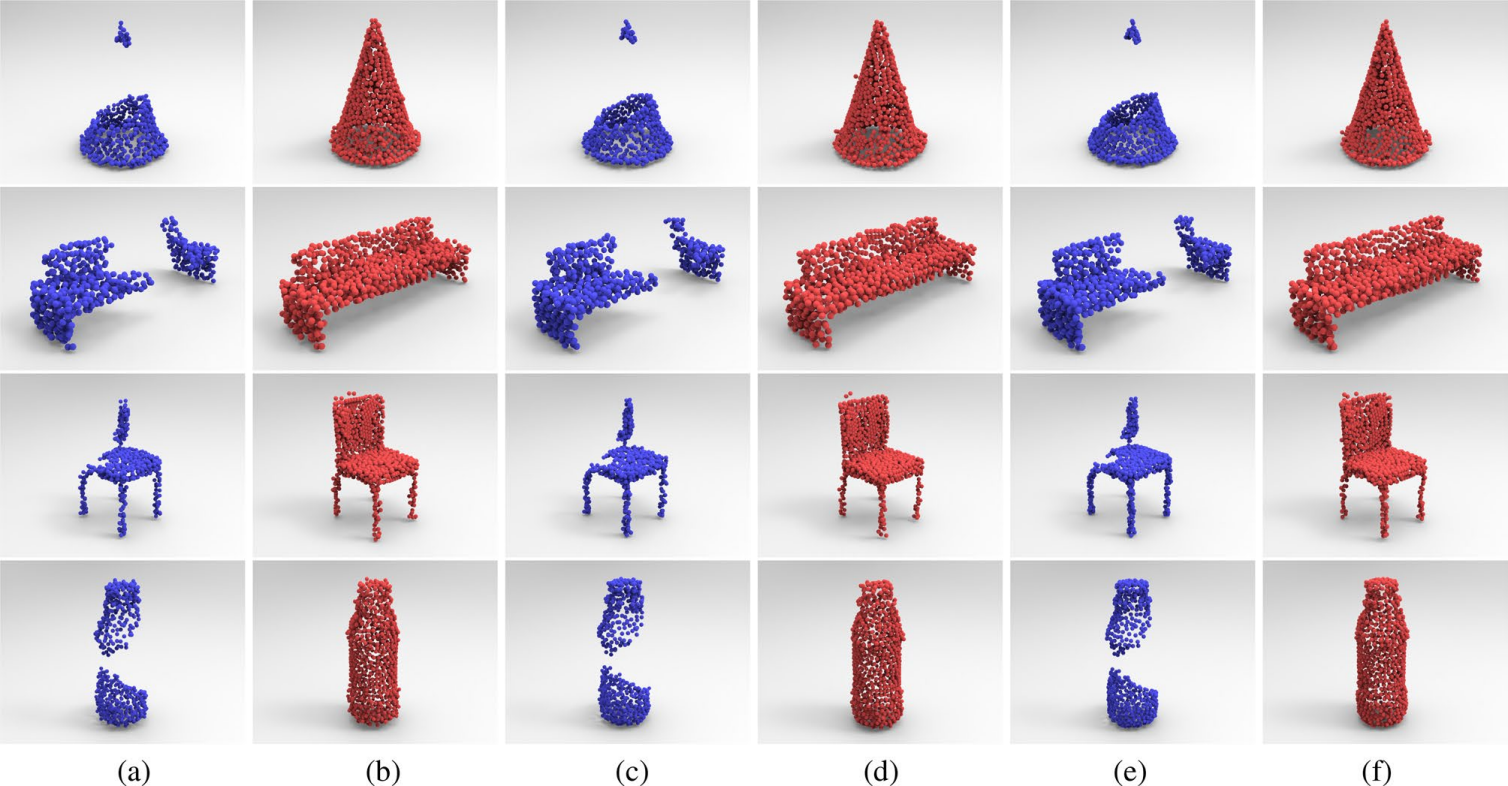
Visual performance of completion results with different model noise: (**a**,**b**) list the input point cloud data with Gaussian noise of standard deviations of 0.014 and corresponding completion results. (**c**,**d**) List the input point cloud data with Gaussian noise of standard deviations of 0.010 and corresponding completion results. (**e**,**f**) List the input point cloud data with Gaussian noise of standard deviations of 0.006 and corresponding completion results.

The proposed coarse-to-fine completion network can encode the incomplete point cloud data into latent feature code-word, which can represent the global features of the underlying point cloud shape and thus robust to model noise. To validate its robustness against model noise, we perturbed the input point clouds with Gaussian noise and fed these shapes to our point completion network. Figure 8 gives the completion results via our network DCSE-PCN for different input noises. Figure 8a,c,e show the input incomplete point clouds with Gaussian noise of standard deviations of 0.014, 0.010 and 0.006, respectively, whilst Fig. 8b,d,f list the corresponding completion results using our proposed network respectively, and each shape contains 1024 sampling points. Table 2 gives the error statistics of the completion results for incomplete point clouds with different levels of Gaussian noise. Figure 8 and Table 2 illustrate its robustness to repair the incomplete models with different input model noises.

**Table 2:** Error statistics of point cloud completion with different model noise disturbance.

Error type	Point cloud models	Noise 0.014	Noise 0.010	Noise 0.0060
CD	Cone	0.04853	0.04582	0.04324
	Beach	0.06607	0.05537	0.05319
	Chair	0.05304	0.05126	0.04818
	Bottle	0.05709	0.05241	0.04753
EMD	Cone	0.04811	0.04739	0.04593
	Beach	0.06970	0.06607	0.06415
	Chair	0.09626	0.05675	0.09310
	Bottle	0.05585	0.05143	0.04432

### Comparisons of different point completion methods

In this section, we compare our proposed network DCSE-PCN against the existing four state-of-the-art point cloud completion methods on ShapeNet dataset^38^ and ModelNet dataset^37^, that is, “Network1” (PCN^11^), “Network2” (FoldingNet^12^), “Network3” (Atlas^14^), “Network4” (CRN^15^) and also “Network5” (coarse completion stage of our network). For a fair comparison, we employ the same network training/testing dataset and keep the same training epoch, the same learning rate, and the same batch size. All the input incomplete point clouds are 2048 sampling points, whilst both the complete point clouds and the ground truth are 2048 sampling points.

**Figure 9:**
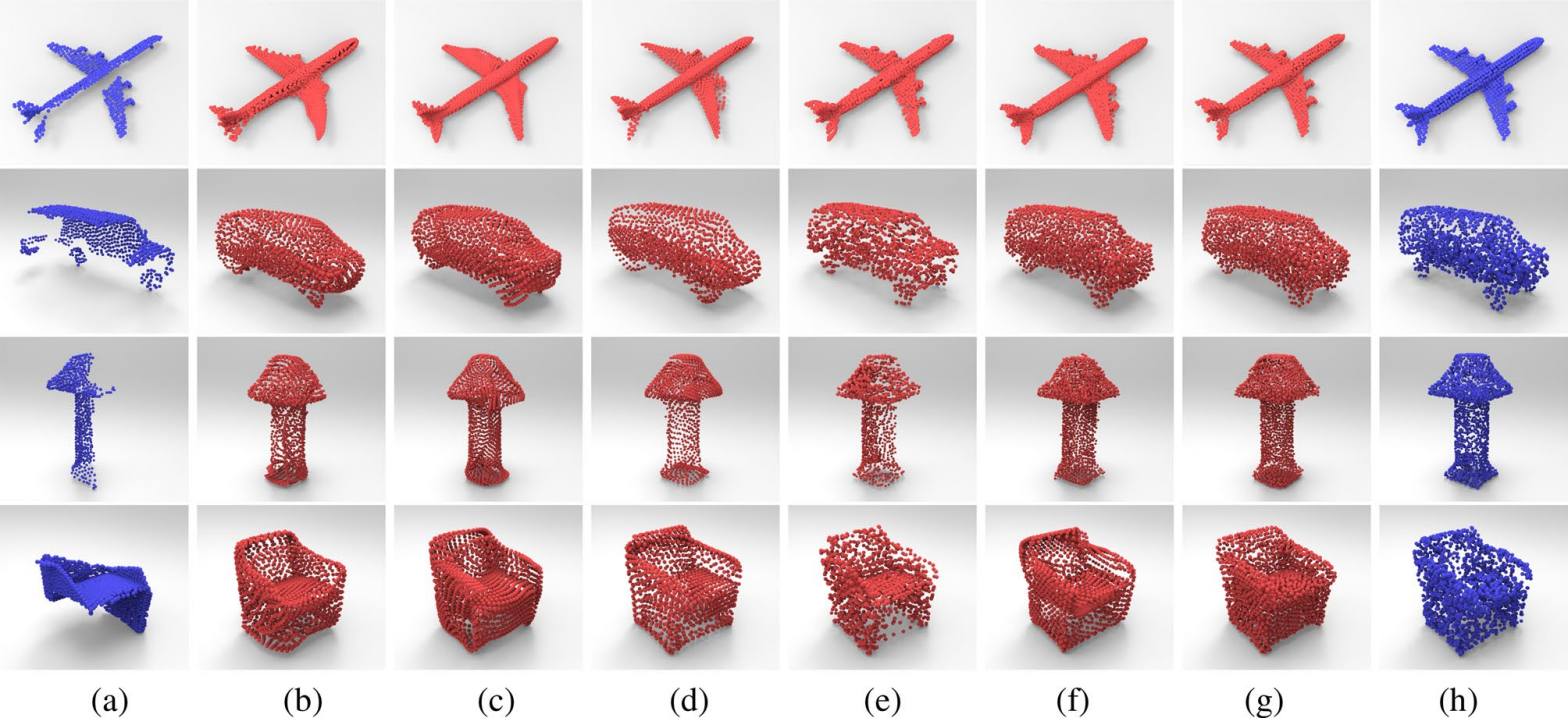
Comparisons of completion results via different methods on ShapeNet dataset^38^: (**a**) input incomplete point cloud. (**b**–**f**) Completion results of “Network1”, “Network2”, “Network3”, “Network4” and “Network5”, respectively. (**g**) Completion results via our proposed network. (**h**) Ground truth.

Figure 9a,h show the input incomplete point clouds and the corresponding ground truth. Figure 9b–f list the completion results of different completion networks respectively, such as “Network1”, “Network2”, “Network3”, “Network4” and “Network5”, whilst Fig. 9g shows the completion results of our proposed coarse-to-fine network. It can be noticed that the completion results of our method are closer to the ground truth for different point cloud shapes due to its effective geometric learning through the modules of local details compensation and shape structure enhancement. For the aircraft model, the completion results using our completion network can effectively recover the shape details of the original shape and also maintain its regular arrangement or symmetric parts, whilst “Network1” and “Network2” could not recover the surface details of the engine of aircraft significantly. The sampling points of the complete car and lamp models repaired by “Network3” and “Network4” are always nonuniform distribution, such as the sticking or uneven spacing between sampling points. However, compared with “Network5”, the final complete car and lamp models generated by our proposed two-stage network always have uniform sampling points and sharp edges thus preserving the shape details. Table 3 reports the data statistics of CD and EMD error for different point cloud models listed in Fig. 9 using different shape completion methods on the ShapeNet dataset^38^. For these distance errors, the smaller value indicates that the recovered shape is closer to the ground truth. From Table 3, we can notice that the CD and EMD error of our proposed network are smaller than other existing networks on most of point cloud models, such as, the repairing results of our proposed two-stage point completion network are reduced by an average of $35.62\%$, $33.31\%$, $29.62\%$, and $23.62\%$ in terms of CD error, comparing with PCN^11^, FoldingNet^12^, Atlas^14^, and CRN^15^ methods, respectively; and also reduced by an average of $15.63\%$, $1.29\%$, $64.52\%$, and $62.87\%$ in terms of EMD error, respectively. The extensive experiments demonstrate that our proposed coarse-to-fine completion network can effectively maintain both the overall shape and local details of the underlying shapes and also show its competitive performance on both CD and EMD distance error, which outperform other state-of-the-art point completion networks.

**Table 3:** Error statistics for different networks on the ShapeNet dataset^38^.

Error type	Point cloud models	PCN^11^	FoldingNet^12^	Atlas^14^	CRN^15^	Coarse stage	Fine stage
CD	Airplane	0.01847	0.01818	0.01824	0.01417	0.01380	**0.01104**
	Car	0.04004	0.03867	0.03833	0.03543	0.03524	**0.02728**
	Lamp	0.03337	0.03450	0.02997	0.02951	0.02662	**0.02206**
	Sofa	0.06361	0.05648	0.05302	0.05326	0.05253	**0.04040**
EMD	Airplane	0.04043	**0.02946**	0.07114	0.08317	0.03239	0.03779
	Car	0.05779	0.05351	0.12337	0.13157	0.04623	**0.04120**
	Lamp	0.04666	0.04004	0.15360	0.14627	0.04620	**0.03894**
	Sofa	0.07455	0.07207	0.22130	0.14742	0.07076	**0.06655**

^∗^Boldface indicates the best value for each evaluation, while the underline indicates the second best.

**Figure 10:**
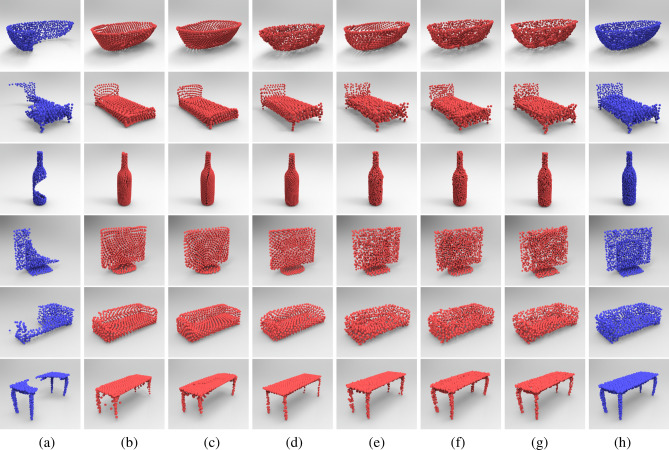
Comparisons of completion results via different methods on ModelNet dataset^37^: (**a**) input incomplete point cloud. (**b**–**f**) Completion results of “Network1”, “Network2”, “Network3”, “Network4” and “Network5” respectively. (**g**) Completion results via our proposed network. (**h**) Ground truth.

Meanwhile, we also compared our completion network with different networks on ModelNet dataset^37^. For the ModelNet dataset, all the input incomplete point clouds are 614 sampling points, whilst the complete point clouds and the ground truth are 1024 sampling points. As shown in Fig. 10, the completion results of our proposed coarse-to-fine network are always regular and can maintain its symmetric parts, whilst the results using “Network2” and “Network3” may lead to some unexpected distortion (e.g., the margin of monitor and sofa, etc). We can also notice that the completion results of our proposed network will always contain less data noise, whilst the completion results using “Network1” and “Network4” will lead to a certain amount of artificial noise (e.g., around the legs of table and the back of bed). Figure 10a,h show the input incomplete point cloud and the corresponding ground truth respectively, from which we can see that the completion results obtained from our coarse-to-fine completion network (see Fig. 10g) are closer to the ground truth. The extensive experiments can also validate the effectiveness of our proposed point completion network for maintaining the overall shape and local details of the complete shapes. Table 4 gives the error statistics of the CD and EMD errors via different completion networks for different models listed in Fig. 10. The experiments show that the CD error and EMD error of our presented coarse-to-fine completion network are smaller than other existing networks on most of the point cloud models, such as, the repairing results of our two-stage point completion network have an average reduction of $28.41\%$, $26.57\%$, $20.65\%$, and $18.55\%$ in terms of CD error, comparing to PCN^11^, FoldingNet^12^, Atlas^14^, and CRN^15^, respectively; and also an average reduction of $21.91\%$, $19.59\%$, $43.51\%$, and $21.49\%$ in terms of EMD error, respectively. The completion results of our proposed network are always closer to that of the ground truth, which shows its technical superiority using our two-stage scheme.

**Table 4:** Error statistics for different networks on the ModelNet dataset^37^.

Error type	Point cloud models	PCN^11^	FoldingNet^12^	Atlas^14^	CRN^15^	Coarse stage	Fine stage
CD	Bottle	0.04265	0.04156	0.03910	0.03972	0.03458	**0.03244**
	Bed	0.06263	0.05992	0.05747	0.05558	0.04676	**0.04481**
	Monitor	0.06156	0.06387	0.05935	0.06250	0.04835	**0.04591**
	Sofa	0.07482	0.06978	0.06471	0.05825	0.05771	**0.05399**
	Table	0.04724	0.04564	0.04095	0.03862	0.03527	**0.02789**
	Bathtub	0.05609	0.05518	0.04948	0.04851	0.04894	**0.04270**
EMD	Bottle	0.04983	0.05039	0.04958	0.04350	0.04310	**0.04027**
	Bed	0.07221	0.06203	0.11517	0.08650	0.06170	**0.05132**
	Monitor	0.11345	0.10843	0.11422	0.07163	**0.06441**	0.06498
	Sofa	0.07530	0.07293	0.12651	0.08745	0.06686	**0.06607**
	Table	0.07158	0.07468	0.11114	0.06422	0.07039	**0.05500**
	Bathtub	0.06038	0.05987	0.10490	0.08510	0.05732	**0.05725**

^∗^Boldface indicates the best value, underline means the second best value.

## Conclusion

To effectively utilize the shape detail information of the input incomplete point cloud, we propose a coarse-to-fine completion network based on details compensation and structural enhancement of the underlying 3d shapes. The network consists of two stages for the task of point cloud completion. The coarse completion stage consists of two branches—shape structure recovery branch and local details compensation branch, where the input is the incomplete point cloud data and the output is a coarse recovered point cloud. The fine completion stage uses a self-attentive mechanism to learn the correlation between neighboring points and combines the coarse completion result with the original point cloud, resulting in a refined completion result with uniform distributed sampling points. Our point completion network is robust to different degrees of data incompleteness and model noise. The quantitative and qualitative results can validate the effectiveness of our coarse-to-fine completion network.

In the future, we could consider the lightweight completion network to achieve robust completion results for highly complex point cloud scene data.

## Data Availability

The datasets analyzed during the current study are available at ShapeNet dataset [https://shapenet.org/] and ModelNet dataset [https://modelnet.cs.princeton.edu/].
